# Evaluation of antibacterial effects of *Zataria multiﬂora* Boiss extracts against ESBL-producing *Klebsiella pneumoniae* strains

**Published:** 2016

**Authors:** Masoud Dadashi, Ali Hashemi, Gita Eslami, Fatemeh Fallah, Hossein Goudarzi, Soroor Erfanimanesh, Arezou Taherpour

**Affiliations:** 1 *Department of Microbiology, School of Medicine, Shahid Beheshti University of Medical Sciences, Tehran, Iran *; 2 *Pediatric Infectious Research Center, Mofid Children Hospital, Shahid Beheshti University of Medical Sciences, Tehran, Iran*; 3 *Department of Microbiology, Kurdistan University of Medical Sciences, Sanandaj, Iran *

**Keywords:** *Klebsiella pneumoniae*, *Extended-Spectrum-β-Lactamases (ESBLs)*, *Zataria multiﬂora*, *Antibiotic Resistance*

## Abstract

**Objective:**

There are few therapeutic options for treatment of multidrug resistant *Klebsiella pneumoniae* isolates as a hospital infectious agent (nosocomial infection). The aim of this study was to evaluate the antibacterial activity of *Zataria multiﬂora *Boiss extracts against ESBL-producing *Klebsiella pneumoniae *strains.

**Materials and Methods:**

This study was conducted on 100 *K. pneumoniae *isolates from two hospitals in Tehran, Iran. Antibiotic susceptibility tests were performed by Kirby-Bauer disc diffusion and microdilution broth methods and detection of ESBL was carried out according to CLSI guidelines. The *bla*CTX-M-15 plasmid gene was detected by PCR and sequencing methods. Extracts susceptibility test was performed by broth microdilution method.

**Results:**

Among 100 *K. pneumoniae *strains, 48 (48%) were ESBL positive. In this study, fosfomycin, colistin and tigecycline were more active than other antibiotics. The existence of *bla*CTX-M-15 was detected in 30 (62.5%) of 48 ESBL-producing isolates. The chloroformic extract showed potent activity against ESBL-producing* K. pneumoniae* strains (MIC_50 _= 1.56 mg/ml and MIC_90_=3.12mg/ml). The MIC_50_ and MIC_90 _(The MIC_50_ represents the MIC value at which ≥50% of the isolates in a test population are inhibited and the MIC_90_ represents the MIC value at which ≥90% of the strains within a test population are inhibited) were 3.12 and 6.25 mg/ml and 6.25 and 12.5 mg/ml for methanolic and acetonic extracts, respectively.

**Conclusion:**

The incidence of ESBL-producing *K. pneumoniae *is very high. Therefore, detection of ESBL-producing *K. pneumoniae *isolates is of great importance in identifying drug resistance patterns in *K. pneumoniae* isolates and in control of infections. *Zataria multiflora *may have the potential to be used against multidrug resistant organisms such as clinical isolates of ESBL-producing *K. pneumoniae*.

## Introduction

In the last decades, Extended-Spectrum-β-Lactamases (ESBLs) in Gram negative bacilli have appeared as a significant mechanism of resistance to antibiotics (Bradford, 2001; Pagani et al., 2000; Sirot et al., 1987). The genes encoding ESBLs are usually found on plasmids, along with genes encoding mechanisms of resistance to cephalosporins, aminoglycosides and other antibiotics. *Klebsiella pneumoniae *isolates harboring ESBLs are significantly more frequently found to be resistant to quinolones than non-ESBL-producing strains (Khayr et al., 2000; Martinez-Martinez et al., 1999). Most commonly, ESBLs derive from genes for TEM-1, TEM-2 or SHV-1 β-lactamases by mutations that modify the amino acid structure around the active site of these enzymes (Nukaga et al., 2003). *K. pneumoniae *may lead to blood stream infections, community and ventilator-acquired pneumoniae (VAP), urinary tract, intra-abdominal pathologies, and central venous line-related infections. *K. pneumoniae *is among the most important causes of both hospital and community-acquired serious bacterial infections in humans (Orencia et al., 2001). 

Before introducing antibiotics and other modern pharmaceuticals, natural products had been used as a traditional medicine. Some plants have incredible effects in treatment of infectious diseases. Therefore, local communities use about 10% of all plants to treat numerous infections. However, less than 1% of them were documented by modern scientists. The popularity of these plants is a trigger to determine antimicrobial substances and their efficacy to treat infectious diseases (Betoni et al., 2006). Plants are rich in an extensive variety of secondary metabolites such as tannins, alkaloids and flavonoids which have been found to have antimicrobial properties, *in vitro* (Lewis and Ausubel, 2006). A number of phytotherapy manuals have suggested various medicinal plants for treating infectious diseases due to their fewer side effects and lower toxicity (Lee et al., 2007). There are several reports about the antimicrobial activity of herbal extractions. According to WHO reports, the best source to obtain drugs are medicinal plants (Al Akeel et al., 2014). Then, it is thoroughly clarified that plant product recognition is very important to find their possible therapeutic properties (Cowan, 1999). There are few plants that are effective against multidrug resistant bacteria, including the ESBL-producing *Escherichia coli *and *K. pneumoniae *(Devatkal et al., 2013). A member of *Labiatae *family is *Zataria multiflora *Boiss (Avishan-e-Shirazi) that grows wild in central and southern parts of Iran. It is useful as an antiseptic, analgesic and carminative agent (Zomorodian et al., 2011). A number of Gram-positive and Gram-negative bacteria are sensitive to *Z. multiflora *antibacterial activity (Zomorodian et al., 2011). The present study was done to determine antibacterial activity of extracts of *Z. multiflora*on against ESBL-producing *K. pneumoniae* strains. 

## Materials and Methods

**Bacterial isolates**

From October 2012 to May 2013, 100 non-duplicate non-consecutive *K. pneumoniae* that belonged to males 55 (55%), females 45 (45%) were collected from hospitalized patients from Mofid Children and Taleghani Hospitals, Tehran, Iran. The isolates were stored at -20 ^o^c in trypticase soy broth containing 20% glycerol.

**Antimicrobial susceptibility testing**

Antimicrobial susceptibility of each *K. pneumoniae* isolate was determined by the Kirby-Bauer disk diffusion method (Mast Group, Merseyside, UK) on Mueller Hinton agar (Merck, Germany) and interpreted as recommended by Clinical Laboratory Standards Institute (CLSI , 2012) or FDA breakpoints (Tigecycline) guidelines. Disks of penicillins [ piperacillin (PIP, 100 μg), ampicillin (AMP,10 μg)], beta lactam/beta lactamase inhibitor combinations [piperacillin/ tazobactam (PTZ, 100/10 μg )], cephems [ceftazidime (CAZ, 30 μg), cefotaxime (CTX, 30 μg), cefepime (FEP, 30 μg), ceftriaxone (CRO, 30 μg), cefpodoxime (CPD, 30 μg)], monobactams [aztreonam (ATM, 30 μg)], carbapenems [imipenem (IPM, 10 μg), meropenem (MEM, 10 μg), doripenem (DOR, 10 μg), ertapenem (ETP, 10 μg)], aminoglycosides [gentamicin (GEN,10 μg), Amikacin (AK, 30 μg)], Tetracyclines [Tetracycline (TE,10 μg)], Fluoroquinolones [Ciprofloxacin (CIP, 5 μg)], [trimethoprim-sulfamethoxazole (TS, 2.5 μg)], Fosfomycins [fosfomycin/trometamol (FOT, 200 μg) ] and tigecycline (TGC, 15 μg) were used and *Escherichia coli* American Type Culture Collection 25922 was used as a control strain. Minimum Inhibitory Concentration (MIC) was determined by broth microdilution method following CLSI guidelines (CLSI, 2012).

**Phenotypic detection of β-lactamases**

Detection of ESBLs was tested for all the isolates by Combination Disk Diffusion Test (CDDT) containing ceftazidime (CAZ) and cefotaxime (CTX) with CAZ 30µg+CA 10 µg and CTX 30µg+CA 10µg per disc (Mast Group, Merseyside, UK). The zone of inhibitions was compared for the CTX, CAZ discs with that of the CAZ 30µg+ Clavulanic (CA) 10 µg and CTX 30µg+ CA 10 disc. An increase in zone diameter of ≥5mm in the presence of clavulanic acid indicated the presence of ESBL in the test organism. *E. coli* ATCC 25922 and *K. pneumoniae* ATCC700603 were used as negative and positive controls for ESBL production, respectively (CLSI, 2012).

**Detection of resistance gene by PCR**

Plasmids DNA were prepared by Plasmid Mini Extraction Kit (Bioneer Company, Korea). Amplification for CTX-M-15 gene was performed with primers CTX-M-15-F (5′- CGTCGGTGACGATTTTAGCC-3′) and CTX-M-15-R (5′- ACCGTCACGCTGTTGTTAGG -3′). Amplification was carried out with the flowing thermal cycling conditions: 5 min at 94°C and 36 cycles of amplification consisting of 1 min at 94°C, 1 min at 55°C, and 1 min at 72°C, with 5 min at 72°C for the final extension. DNA fragments were analyzed by electrophoresis in a 1% agarose gel at 95 V for 45 min in 1X TBE containing ethidium bromide.

**Sequencing**

The PCR purification kit (Bioneer Co., Korea) was used to purify PCR products and sequencing of forward strand was performed by the Bioneer company (Korea). The nucleotide sequences were analyzed with Chromas 1.45 and MEGA-4 software’s and BLAST in NCBI.

**Plant material**

*Source, Collection and Identification*

During 2012, leaves of *Zataria multiﬂora *Boiss were collected from Fars province in Iran. Voucher specimens (TMRC1429) have been deposited in the herbarium of Traditional Medicine and Materia Medica Research Center (TMRC), Shahid Beheshti University of Medical Sciences, Tehran, Iran.

**Extraction method**

Leaves of the *Zataria multiﬂora *Boiss plant (400g) were dried at 25^o^C and then powdered using a mechanical grinder. Ten grams of dried plant was soaked in 100 ml methanol (96%, v/v), acetone (99% purity) and chloroform (99.4**% **purity) (Merck, Germany) separately for a period of 48 hours without any heating procedure. Each extract was first filtered through a Whatman No. 1 filter paper and then through a 0.45 µm membrane filter. The filtrate was evaporated under reduced pressure in vacuum evaporator and stored at 4^o^C. After drying, extracts were stored at -20ºC.

**Determination of MIC by broth Microdilution**

The minimum inhibitory concentration (MIC) of the extracts was determined (CLSI, 2012). *Zataria multiﬂora *Boiss extracts were diluted with 2% DMSO to yield concentrations ranging from 25 to 0.19 mg/ml. Cation-adjusted Muller Hinton broth was used as broth medium. After shaking, 0.1 ml of each extract was added to each well of 96-well microtiter plates. Microbial suspensions were adjusted to 0.5 MacFarland and diluted 1:10 to yield 1×10^7^ CFU/ml and to each well, 0.005 ml of the bacterial inoculum was seeded. Control line with no bacterial inoculation and *Escherichia coli* ATCC 25922 were simultaneously maintained. Microplates were incubated aerobically at 37°c for 18-24 hours. The lowest concentration of the extracts that showed no visible bacterial growth was reported as the MIC.

## Results

**Figure 1:**
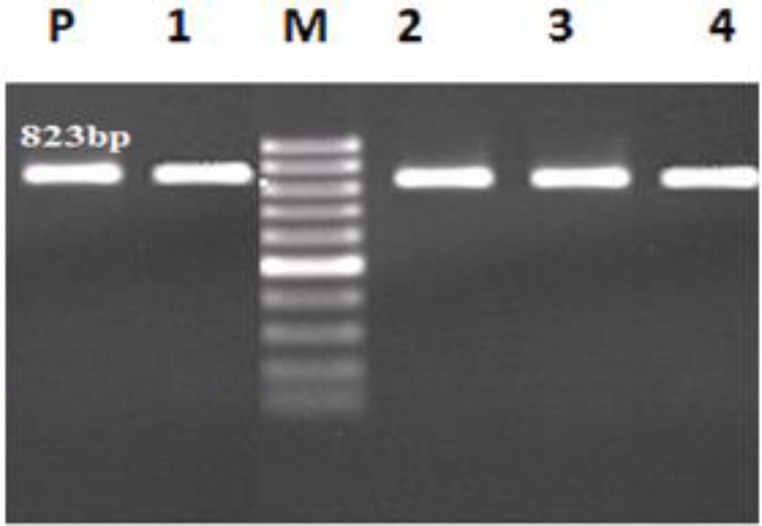
PCR amplification of CTX-M-15 gene. Lane M: DNA size marker. Lane P: Positive control. Lane 1,2,3 and 4; CTX-M-15 (213bp), genes positive isolates

Among the 100 *K. pneumoniae* strains, 48 (48%) were ESBL positive. The lowest rates of resistance in isolates were observed for fosfomycin 3 (3%), tigecycline 5 (5%) and colistin 0 (0.0%) (Table 1). The existence of *bla*CTX-M-15 was detected in 30 (62.5%) of 48 ESBL-producing isolates (Figure1). MIC results for studied isolates are shown in Table 2. The chloroformic extract showed higher activity against ESBL-producing* K. pneumoniae* strains (MIC_50 _= 1.56 mg/ml and MIC_90_=3.12mg/ml). The MIC_50_ and MIC_90 _were 3.12 mg/ml and 6.25 mg/ml and 6.25 mg/ml and 12.5 mg/ml for methanolic and acetonic extracts, respectively. 

**Table 1:** Antimicrobial drug–resistance patterns of 100 *K. pneumoniae* isolates

**Antibiotic**	**Resistant** **No (%)**	**Intermediate** **No (%)**	**Sensitive** **No (%)**
**Aztreonam (10 μg)**	56	8	36
**Meropenem (10 μg)**	20	10	70
**Gentamicin(10 µg)**	36	4	60
**Ciprofloxacin (30 μg)**	53	7	40
**Amikacin (30 µg)**	26	4	70
**Imipenem(10 μg)**	20	10	70
**Cefotaxime(30 µg)**	57	3	40
**Cefepime(FEP, 30 μg)**	37	10	53
**Tetracycline(TE,10 μg)**	50	7	43
**Ampicillin(AMP,10 μg)**	73	7	20
**Piperacillin(PIP,100 μg)**	57	3	40
**Ceftriaxone(CRO,30 μg)**	56	4	40
**Cefpodoxime(CPD,30μg)**	64	6	30
**Tigecycline(TGC,15μg)**	5	30	65
**Doripenem(DOR,10 μg)**	20	10	70
**Ertapenem(ETP, 10 μg)**	20	10	70
**Piperacillin/Tazobactam (PTZ,100/10μg)**	29	10	61
**Fosfomycin/Trometamol (FOT, 200 μg)**	3	12	85
**Ceftazidime (30 µg)**	54	6	40
**Colistin (C0,10** ** µg)**	0	0	100

**Figure 2:**
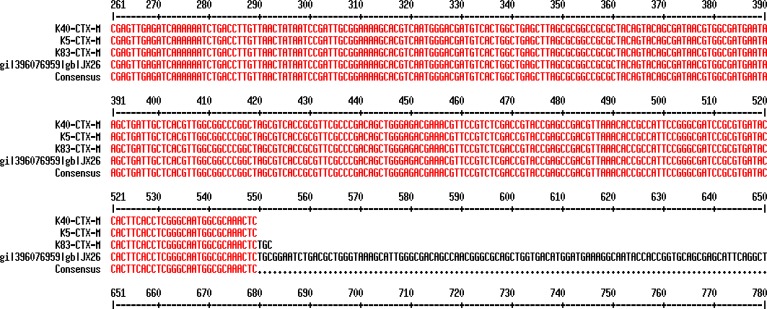
Multiple Sequence Alignment

The MIC_50_ represents the MIC value at which ≥50% of the isolates in a test population are inhibited and the MIC_90_ represents the MIC value at which ≥90% of the strains within a test population are inhibited. The nucleotide sequence data reported in this paper have been submitted to the GenBank sequence database and assigned accession number KF513160. The sequences of *bla*CTX-M-15 gene in *K. pneumoniae* strains isolated from Mofid Children and Taleghani Hospitals were the same (http://multalin.toulouse.inra.fr/multalin/cgi-bin/multalin.pl) (Figure 2).

**Table 2:** Minimum inhibitory concentrations of different antibiotics (µg/ml) and extracts (mg/ml) against the 100 *K. pneumoniae* isolates

**Antibiotics and extracts**	**MIC**		
	**Range**	**50%**	**90%**
**Meropenem**	0.25-256	1	32
**Imipenem**	0.25-256	1	16
**Ceftazidime**	1->256	64	>256
**Ceftriaxone**	0.5->256	16	>256
**Cefepime**	0.5->256	16	>256
**Cefotaxime**	0.5->256	16	>256
**Piperacillin/Tazobactam**	0.25-256	4	128
**Ampicillin**	2->256	256	>256
**Ciprofloxacin**	0.5->256	16	>256
**Chloroformic **	0.39-6.25	1.56	3.12
**Methanolic **	0.78-12.5	3.12	6.25
**Acetonic**	078-25	6.25	12.5

## Discussion

Antibiotic resistance among Gram-negative bacteria is an emerging concern of signiﬁcant clinical and public health importance worldwide. The threat caused by *K. pneumoniae *in hospital-borne infections and the need for an effective antibiotic led us to search for a potential anti-infective agent against this pathogen. Therefore, herbal evaluation is one step toward achieving the natural antibiotic for controlling and treating infection without appearance of resistant strain. In this study, the lowest rates of resistance in isolates were observed for fosfomycin 3 (3%), tigecycline 5 (5%), amikacin 12 (12%), ertapenem 20 (20%), doripenem 20 (20%), meropenem 20 (20%), imipenem 20 (20%) and piperacillin/tazobactam 29 (29%). The highest rates of resistance were observed for ampicillin 73 (73%), cefpodoxime 64 (64%), piperacillin 50 (50%), cefotaxime 57 (57%), aztreonam 56 (56%), ceftriaxone 56 (56%), ceftazidime 54 (54%) and ciprofloxacin 53 (53%). So, the best coverage against the studied isolates was obtained with fosfomycin , colistin and tigecycline. The increasing rate of ESBL production among *K. pneumoniae *clinical isolates is perturbing and the fact that most of these organisms are multidrug resistant brings serious concern. Recently, CTX-M-15-producing *K. pneumoniae *has disseminated worldwide (Shin et al., 2006). The *bla*CTX-M-15 gene was detected in 30 (62.5%) of 48 ESBL-producing isolates and all were resistant to ceftazidime and cefotaxime. Only a few antibacterial drugs were effective against the ESBL-producing *K. pneumoniae *that were isolated from Mofid Children and Taleghani hospitals. Hence, control and treatment of these infections caused by the above-mentioned bacteria is difficult and treatment protocols to prevent resistant genes spread among clinical isolates should be revised. The antimicrobial activity of *Z. multiﬂora* has been shown in many studies but antibacterial effects of these plants against ESBL-producing *K. pneumoniae *strains have not been yet studied. Based on the previous studies, avishen-e-shirazi extract inhibits the growth of *E. coli*, *Salmonella spp*, *Shigella spp*, *Staphylococcus aurous *(Mayrhofer et al., 2004), *Enterococcus* spp. (Mir et al., 1998) and *Pseudomonas aeruginosa* (Salarbashi et al., 2014). Motevasel et al. showed that the alcoholic extract of *Zataria* at low concentrations can inhibit the growth of Gram positive bacteria (Motevasel et al., 2013). Also, Eftekhar reported that *Z. multiflora* essential oil had a considerable *in vitro* activity against the standard ATCC cultures as well as the clinical isolates of *K. pneumoniae *(Eftekhar et al., 2011). In this study, the MIC and MBC values were 0.015- 2.0 mg/ml for ATCC strains and 0.03 to 0.5 mg/ml for the clinical isolates that are lower than what we obtained. The difference between the results may be because essential oils rather than extracts were used in the study done by Eftekhar. Essential oils show considerable antimicrobial and antioxidant activity because of their carvacrol and thymol content (Eftekhar et al., 2011). In comparison, *Z. multiflora* extract is rich in rosmarinic acid that has been shown to have antimicrobial properties. Plant extracts are also easier and cheaper to get. In 2010, Saei-Dehkordi et al. reported that *P. aeruginosa* growth can be inhibited by 2-8 mg/ml of *Z. multiﬂora *Boiss essential oil (Saei-Dehkordi et al., 2010). Furthermore, Mahboubi et al. showed that *staphylococcus *growth can be inhibited by 0.5-1 µl/ml of *Z. multiﬂora *Boiss essential oils (Mahboubi et al., 2010). In our study, we found that the *Z. multiflora *plant extracts had high antibacterial activity against all ESBL-producing *K. pneumoniae *strains isolated from patients and its effective components may provide an alternative treatment for infections caused by multidrug resistant ESBL-producing *K. pneumoniae*. Further studies about the isolation of active compounds and the probable toxicity of plant extracts are necessary to make it as an alternative choice against resistant species. The results of the present study showed that *Z. multiflora *plant extracts had high antibacterial activity against all ESBL-producing *K. pneumoniae *strains isolated from patients in comparison with conventional antibiotics, *in vitro*. We hope that the results of this study contribute to further use of medicinal plants as effective treatments for infections caused by multidrug resistant, beta-lactamase producing *K. pneumoniae*.
